# Dual-energy CT virtual non-calcium: an accurate method for detection of knee osteoarthritis-related edema-like marrow signal intensity

**DOI:** 10.1186/s13244-023-01407-8

**Published:** 2023-04-30

**Authors:** Heng Zhao, Hui Li, Xia Xie, Hai-yan Tang, Xiao-xin Liu, Yi Wen, Xin Xiao, Lu Ye, You-wei Tang, Gao-yue Dai, Jia-ni He, Li Chen, Qian Wang, De-qiu Tang, Shi-nong Pan

**Affiliations:** 1 The First Affiliated Hospital, Department of Radiology, Hengyang Medical School, University of South China, Hengyang, 421001 Hunan China; 2 Department of Radiology, Shengjing Hospital of China Medical University, Sanhao Street No. 36, Heping District, Shenyang, 110004 Liaoning China; 3 Department of Radiology, The First People’s Hospital of Zhaoqing City, Zhaoqing, China

**Keywords:** Osteoarthritis, Edema like marrow signal intensity, Virtual non-calcium, MRI

## Abstract

**Objectives:**

To evaluate the performance of a dual-energy computed tomography (DECT) virtual non-calcium (VNCa) technique in the detection of edema-like marrow signal intensity (ELMSI) in patients with knee joint osteoarthritis (OA) compared to magnetic resonance imaging (MRI).

**Methods:**

The study received local ethics board approval, and written informed consent was obtained. DECT and MRI were used to examine 28 knees in 24 patients with OA. VNCa images were generated by dual-energy subtraction of calcium. The knee joint was divided into 15 regions for ELMSI grading, performed independently by two musculoskeletal radiologists, with MRI as the reference standard. We also analyzed CT numbers through receiver operating characteristics and calculated cut-off values.

**Results:**

For the qualitative analysis, we obtained CT sensitivity (Readers 1, 2 = 83.7%, 89.8%), specificity (Readers 1, 2 = 99.5%, 99.5%), positive predictive value (Readers 1, 2 = 95.3%, 95.7%), and negative predictive value (Readers 1, 2 = 97.9%, 98.7%) for ELMSI. The interobserver agreement was excellent (κ = 0.92). The area under the curve for Reader 1 and Reader 2 was 0.961 (95% CI 0.93, 0.99) and 0.992 (95% CI 0.98, 1.00), respectively. CT numbers obtained from the VNCa images were significantly different between regions with and without ELMSI (*p* < .001).

**Conclusions:**

VNCa images have good diagnostic performance for the qualitative and quantitative analysis of knee osteoarthritis-related ELMSI.

## Key points

- DECT-VNCa images had important clinical significance in diagnosing ELMSI.
- DECT-VNCa images can improve the diagnosis of ELMSI by cut-off value.
- The research of DECT-related post-processing technology in the diagnosis of OA-related pain needs further study.

## Background

The most common type of arthritis worldwide is osteoarthritis (OA), and it is the main cause of dysfunction and disability in the elderly [1–3]. The knee joint is most commonly involved, and the estimated lifetime risk of knee OA is about 45% according to the Johnston County OA Project [1, 4]. Knee OA-related knee pain is common and is a frequent contributor to decreased quality of life in those affected. However, the underlying mechanism of knee pain in OA remains poorly understood [4–6].

Cartilage loss is a characteristic pathological feature of OA, although it is generally recognized that OA is a disease of the whole joint, involving the bone subchondral bone complex, and local soft tissues including synovium, meniscus, and ligament can also undergo pathological changes [1, 6, 7]. Recent studies suggest a strong association between ELMSI and pain in patients with knee OA, new or enlarging OA-ELMSI can be associated with accelerated cartilage loss on MRI when patients were followed up over time. Not surprisingly, given its association with new pain and/or pain intensity, multiple studies have shown that patients with OA-ELMSI are more likely to present for total knee arthroplasty, it can be found that in patients with knee osteoarthritis, the presence of ELMSI is important to guide the clinical evaluation of the cause of pain, and it can support in making the decision concerning appropriate treatment options [8–11]. The pain may be caused by osteoclasts under the subchondral bone reconstruction, especially the growth of nerve axons that dominate OA pain; and it is further suggested that early subchondral bone changes are involved [12–14].

The musculoskeletal system consists of two distinct areas of anatomy: bone and soft tissue. Two of the most valuable methods of analyzing these two major areas are CT and MRI. CT is cheaper and faster than MRI. Moreover, the radiation dose of DECT scan is not significantly higher than that of other CT scans. Subchondral ELMSI on MRI has been highly correlated with pain [13–15]. Clinically, although Dixon technique can quantitatively identify water and fat, some scholars believe that fluid-sensitive MRI sequences are a non-invasive technique for identifying ELMSI [16–19]. However, some patients cannot undergo MRI for various reasons, making it unsuitable for the evaluation of acute knee pain [20, 21]. DECT uses two X-ray tubes operating at different tube voltages at the same to deploy two different X-ray energy spectra and show different tissue characteristics. The VNCa technology uses bone mineral, yellow bone marrow and red bone marrow absorption curves on the two DECT X-ray spectra, which allows calcium reduction through image post-processing [22–24]. We hypothesized that DECT VNCa could achieve good consistency in detecting knee OA-related ELMSI compared with MRI. The purpose of our study was to determine the diagnostic accuracy of VNCa post-processing technology to detect knee OA-related ELMSI.

## Materials and methods

This prospective study (Clinical Trial Registration No. ChiCTR1900024305) was approved by the local ethics committee, and written informed consent was obtained from all participants.

### Study participants

**Fig. 1:**
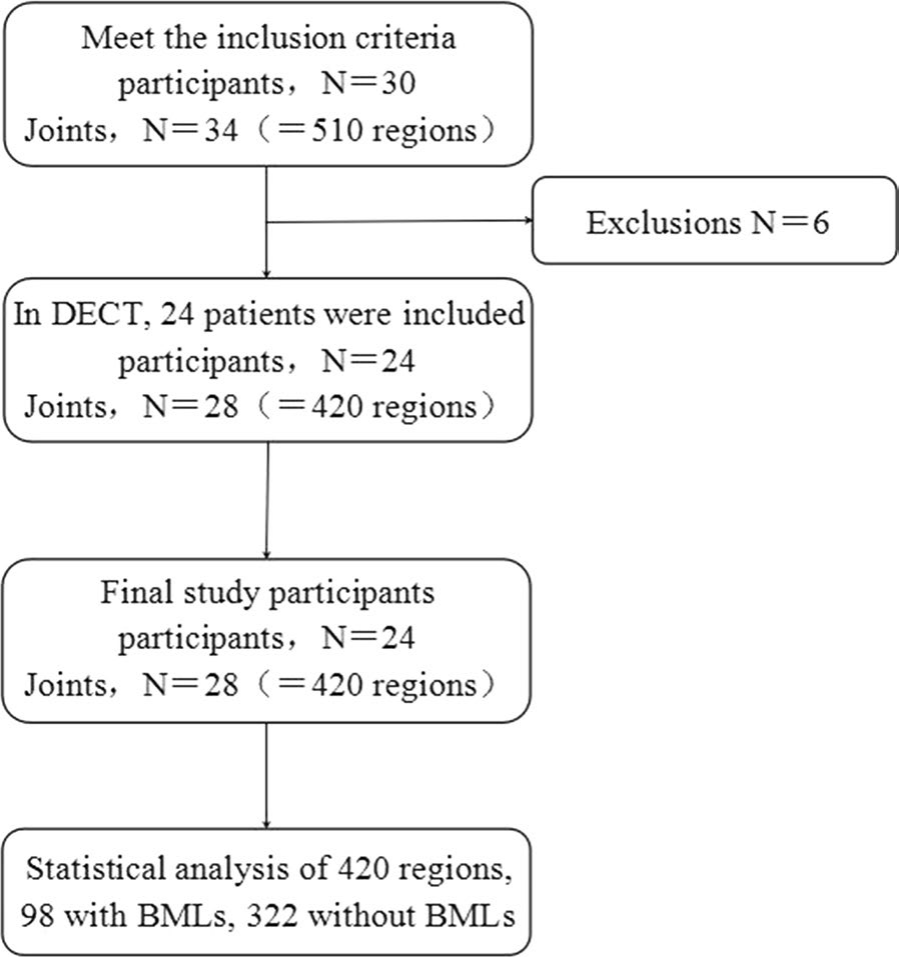
Flow diagram of the study participant selection

Between August 2017 and November 2018, 30 patients (11 men and 19 women) underwent DECT of the knee(s) within 24 h after MRI. After, 6 patients were excluded (1 case of bone infarction, 1 case of rheumatoid arthritis, 2 cases of bone tumor-like lesions, and 2 cases of substandard image quality), 420 regions of 28 knee joints were included in the analysis (Fig. 1). The diagnostic criteria were recurrent episodes of knee arthralgia for one month [25]; X-ray, CT, and MRI examination showing asymmetric joint space narrowing, subchondral bone sclerosis and/or cystic degeneration, and osteophytes of the joints; clear and viscous synovial fluid (at least 2 times) with leukocyte counts less than 2000/-mL; a duration of morning stiffness less than 30 min; bony crepitus occurring with joint activity; normal or slightly elevated ESR or CRP levels [25–29]. The exclusion criteria included patient age < 40 years; diagnosis of axial spondyloarthritis, rheumatoid arthritis, reactive arthritis, psoriatic arthritis, or any other type of chronic immune disease; systemic corticosteroid therapy; contraindications to MRI; and refusal of participation or to participate, recently [30–34].

### DECT scanning parameters (image reconstruction) and post-processing data

All the participants in this study underwent DECT scanning (Somatom Definition Flash, Siemens Healthineers). The scanner had two different kilovoltage X-ray tubes: low (A tube) and high (B tube). The tube voltage was set to 80 and 140 kVp, respectively. All scans were performed in the caudal-cranial direction with patients in a supine position. The acquisition parameters were employed (collimation, 40 × 0.6 mm; pitch, 0.7; rotation time, 1.0 s). We assumed a 2:1 ratio of the tube current–time product (A tube, 360 mAs; B tube, 180 mAs). The calculated mean volume CT dose index of each parameter based on our scan parameters was 12.9 ± 2.5 mGy (range, 11.3–16.3 mGy), and the mean dose length product was 287.8 ± 178.2 mGy·cm (range, 115.6–537.8 mGy·cm). In the default case of the CT scan, the DECT scan can obtain three different sets of images: 80 kVp, Sn140 kVp, and weighted average. We simulated the weighted average with a ratio of 0.3:0.7 to obtain a standard CT image with a contrast of 120 kVp. For post-processing data, our algorithm reconstructed axial slices of the 80- and Sn140 kVp datasets with a section thickness of 0.6 mm (0.5 mm increments).

We used commercial software (Syngo MMWPVE40B Siemens) to process the CT image post-processing data. Based on our previous experience, we set the relative contrast ratio to 1.56 at 80- and Sn140 kVp. The intensity of the smoothing filter range was set to 4, and the color-coded maps were used for further analysis.

### MRI sequence parameters

MRI was performed with a 3.0-T scanner (Achieva, Philips Healthcare, Best) using a knee coil. The MRI parameters used by the investigators for examining patients with knee OA were: sagittal T1-weighted spin-echo MR sequence (repetition time msec/echo time msec, 633/20; ETL, 6; FOV, 160 mm × 160 mm × 80 mm; acquisition matrix, 384 × 312; section thickness, 2.5 mm; intersection gap, 0 mm), axial T2-weighted fat-saturated fast spin-echo MR sequence (5069/63; ETL, 15; FOV, 160 mm × 160 mm × 80 mm; acquisition matrix, 292 × 216; section thickness, 2.5 mm; intersection gap, 0 mm), sagittal and coronal T2-weighted fat-saturated fast spin-echo MR sequence (4835/65; ETL, 15; FOV, 150 mm × 150 mm × 84 mm/160 mm × 160 mm × 84 mm; acquisition matrix, 272 × 195/308 × 240; section thickness, 3 mm; intersection gap, 0 mm).

### Qualitative analysis

**Fig. 2:**
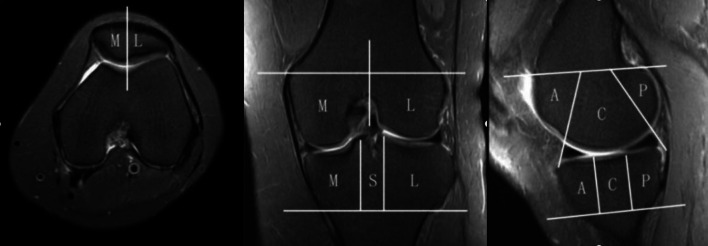
Regional subdivision of the articular surfaces. In the axial plane, the patella (left image) is divided into medial (M) and lateral (L) regions, with the ridge considered part of the M region. In the coronal plane, the femur and tibia are also divided into M and L regions, with the trochlear groove of the femur considered part of the M region. The spines (S) region represents the portion of the tibia beneath the tibial spines. In the sagittal plane, the femoral and tibial surfaces are further subdivided into anterior (A), central (C), and posterior (P) regions (middle image). Region A of the femur corresponding to the patellofemoral articulation; region C to the weight-bearing surface, and region P to the posterior convexity that articulates only in extreme flexion. Region C of the tibial surface corresponds to the uncovered portion between the anterior and posterior horns of the meniscus centrally and the portion covered by the body of the meniscus peripherally

We divided each knee into 15 regions according to anatomical characteristics (Fig. 2). The patella was divided into a lateral facet and a medial facet. The patellar ridge was considered to be part of the medial facet. The femoral articular surface was divided into medial and lateral condyles, and the trochlear groove was a part of the medial condyle. Both medial condyle and lateral condyle were divided into three regions: (1) anterior, from the anterior edge of the superior osteochondral junction to the anterior meniscus angle; (2) center, extending from the leading edge of the anterior horn of the meniscus to the attachment of the posterior capsule of the posterior horn of the meniscus; and (3) posterior, extending from the posterior capsule accessory of the posterior horn of the meniscus to the posterior superior and posterior junction of bone and cartilage. The medial tibial plateau and the lateral tibial plateau were divided into three equal subregions: anterior, middle, and posterior, and the tibial spines were considered a separate region [35, 36].

**Fig. 3:**
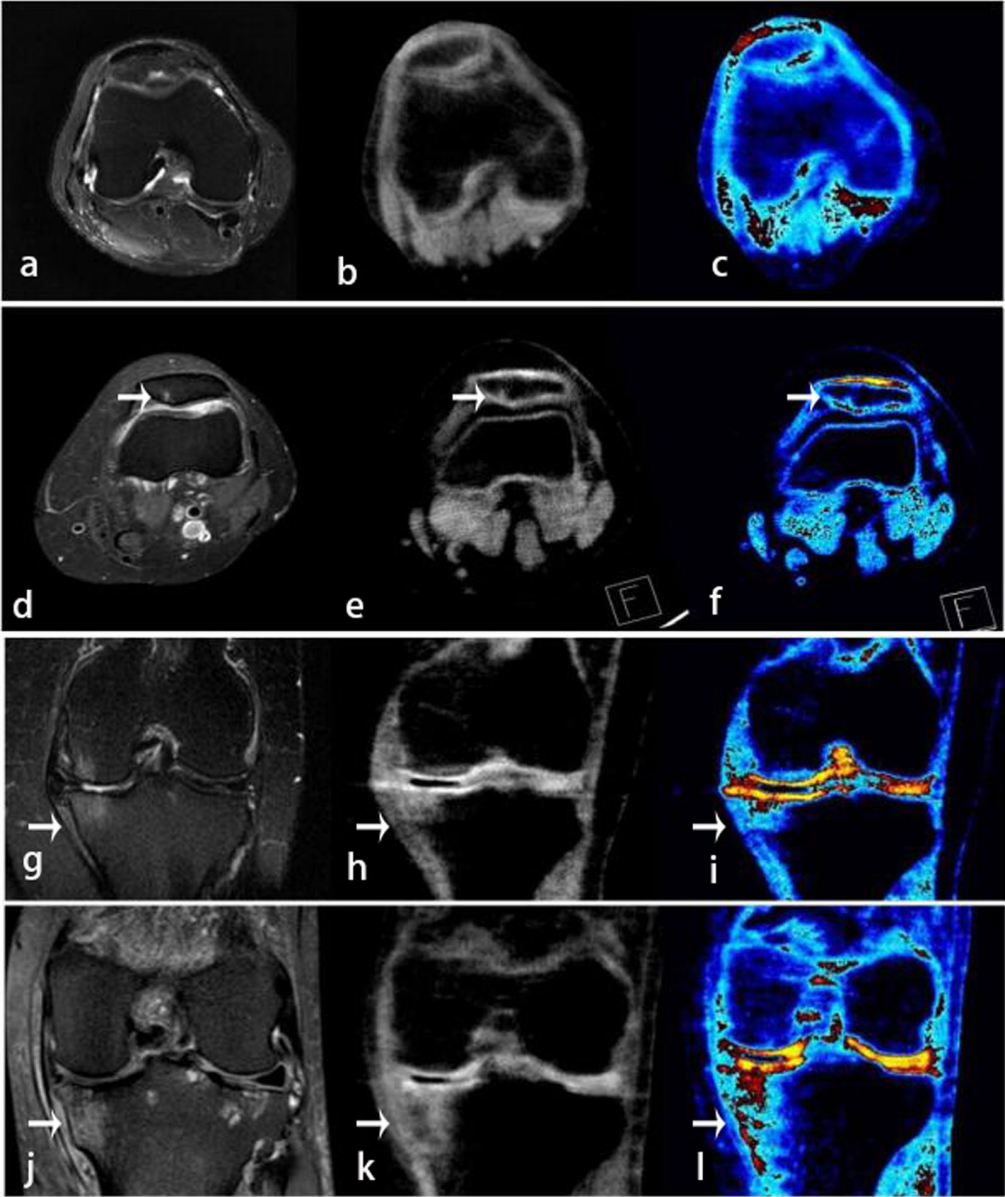
**a**–**c** Axial MRI, DECT VNCa, and color-coded map images showing Grade 1 ELMSI in the patella. **d**–**f** Axial MRI, DECT VNCa, and color-coded map images showing Grade 2 ELMSI in the patella. **g**–**i** Coronal MRI, DECT VNCa, and color-coded map images showing Grade 3 ELMSI in the tibial plateau. **j**–**l** Coronal MRI, DECT VNCa, and color-coded map images showing Grade 4 ELMSI in the tibial plateau (arrow)

The data were analyzed independently by two radiologists (Reader 1 and Reader 2) with 15 and 6 years of experience in musculoskeletal radiology. Both were unaware of the patients’ other imaging data and clinical information. Each reader analyzed the reconstructed and post-processing DECT images. Scoring was based on the severity of the ELMSI in each region of the knee joint as follows: Grade 4, severe ELMSI (showing high density shadow); Grade 3, moderate ELMSI (showing relatively high density shadow); Grade 2, mild ELMSI (showing a slightly higher density shadow); and Grade 1, no ELMSI (showing high density without visible abnormalities). MRI was used as a reference standard to evaluate the severity of ELMSI (Fig. 3). The readers optimized the window settings and size adjustments for diagnosis. We only included ELMSI larger than 2 mm in the subchondral bone to reduce the number of false positive results due to artifacts.

MRI was used as a reference standard to evaluate the severity of ELMSI. A third radiologist (Reader 3) with 18 years of experience in musculoskeletal imaging, who did not know the DECT results, evaluated the MRI for ELMSI. Reader 3 changed the window settings and size adjustments of the MRI as needed.

### Quantitative analysis of CT numbers

**Fig. 4:**
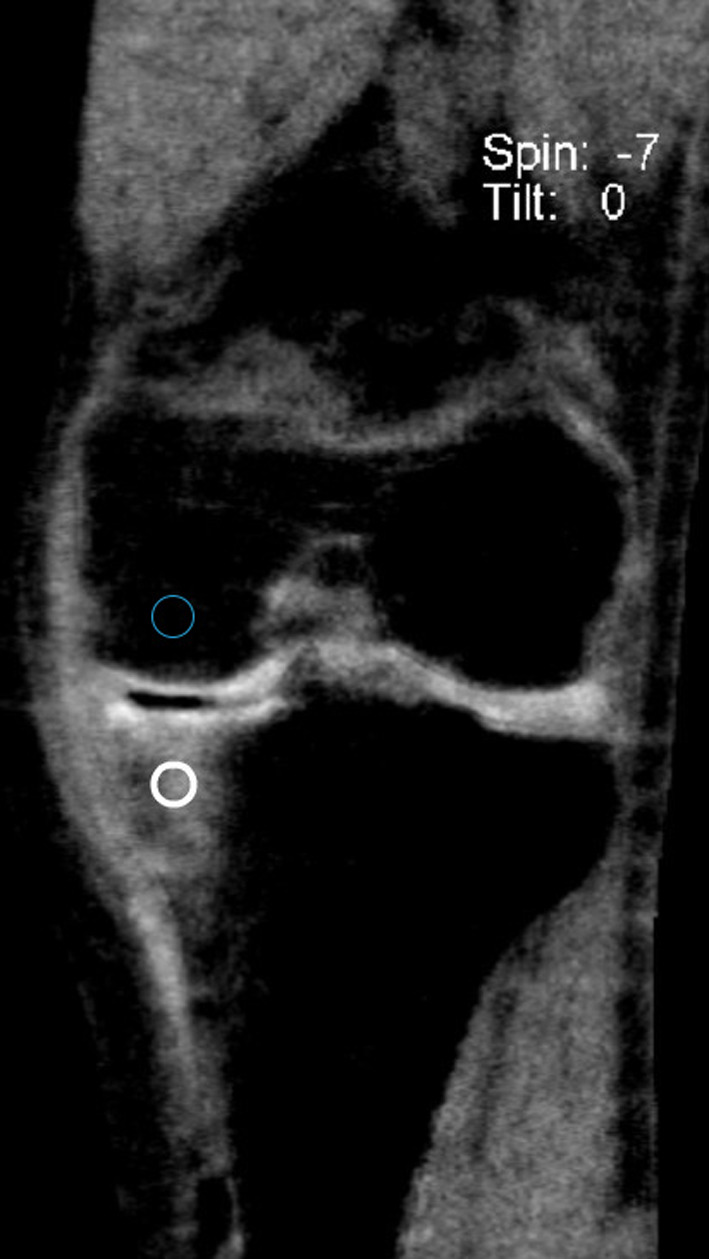
Shows a schematic diagram of ELMSI with DECT VNCa. The blue ROI is in the normal bone marrow area, and the white ROI is in an area of ELMSI

After qualitative identification and grading of ELMSI, the regions were quantitatively analyzed by Readers 1 and 2. The readers used MRI as the reference standard for the presence of ELMSI to measure the CT numbers, independently. In the ELMSI regions of the MRI, we found the corresponding regions in the VNCa image and used circular ROIs to obtain the CT numbers. For each study participant, two circular ROIs in the ELMSI regions of the VNCa image were placed to obtain the DECT VNCa mean CT numbers. Two ROIs at least of 50 mm^2^ were also randomly placed on bone marrow to obtain CT numbers (Fig. 4).

### Statistical analysis

We used Cohen’s Kappa statistic to analyze the inter-reader agreement in the visual assessment of the DECT VNCa images. We used the McNemar test to analyze the visual differences between the two readers for different grades of ELMSI. Using visual analysis data for the DECT VNCa images, we calculated sensitivity, specificity, positive predictive value (PPV, defined as the presence of ELMSI on MRI and the absence of ELMSI on DECT), and negative predictive value (NPV, defined as the presence of ELMSI on DECT and the absence of ELMSI on MRI).

We used a receiver operating characteristic (ROC) curve and its associated statistics to analyze the CT numbers of dual-energy VNCa images. Then, based on the ROC analysis, we compared the CT numbers with the ELMSI grades found in MRI to determine the most accurate cut-off CT numbers of ELMSI. Continuous variables are reported as mean ± standard deviation and were analyzed with the *t* test.

SPSS statistical software was used for analysis. *p* < 0.05 was considered significant.

## Results

A total of 24 participants were included in this study. For a total of 420 region of 28 knee joints for analysis. Study participant statistics are shown in Table 1.

**Table 1:** Study participant statistics

Characteristics of the study	
*Sex*	
Female patients	
No of patients	16
Age (years)	59 (43–76)
Male patients	
No of patients	8
Age (years)	59 (47–66)
*Body mass index (kg/m*^*2*^*)*	24 ± 3
*Bone marrow lesions classified with MRI*	
Grade 1	322
Grade 2	50
Grade 3	16
Grade 4	32

DECT was performed within 24 h after MRI. Of the 510 regions available for analysis, 420 regions (82.4%) were classified as compliant with standards.

### Qualitative analysis of ELMSI identified on VNCa images

In our experiments, we found that the incidence of ELMSI in the medial tibia and femur was the highest, accounting for 4% and 5%, respectively, of which the medial central portions of the femur and tibia were both 2%. Inter-reader agreement for qualitative analysis of ELMSI was very good (*κ* = 0.92). Reader 1 found 49 areas of ELMSI found to be true positives on MRI, while 8 regions were classified as no ELMSI on the DECT VNCa images. Five false negative and two false positive ELMSI results were found by Reader 2. We identified the presence of ELMSI through DECT VNCa images and finally obtained the total sensitivity, specificity, PPV, and NPV (Table 2). In this study, it was found that the sensitivity, specificity, PPV, and NPV of the patella were lower than those in other bones of the knee. This may be related to the small volume of the patella, and therefore, the ELMSI evaluation in this area is affected by the surrounding bone cortex.

**Table 2:** The diagnostic value of VNCa images in ELMSI

Variable	Sensitivity (%)	Specificity (%)	PPV (%)	NPV (%)
Reader 1	83.7 (69.8, 92.2)	99.5 (97.9, 99.9)	95.3 (82.9, 99.1)	97.9 (95.7, 99.0)
Reader 2	89.8 (77.0, 96.2)	99.5 (97.9, 99.9)	95.7 (84.0, 99.2)	98.7 (96.7, 99.5)

Data in parentheses are 95% confidence intervals

### Quantitative analysis

**Fig. 5:**
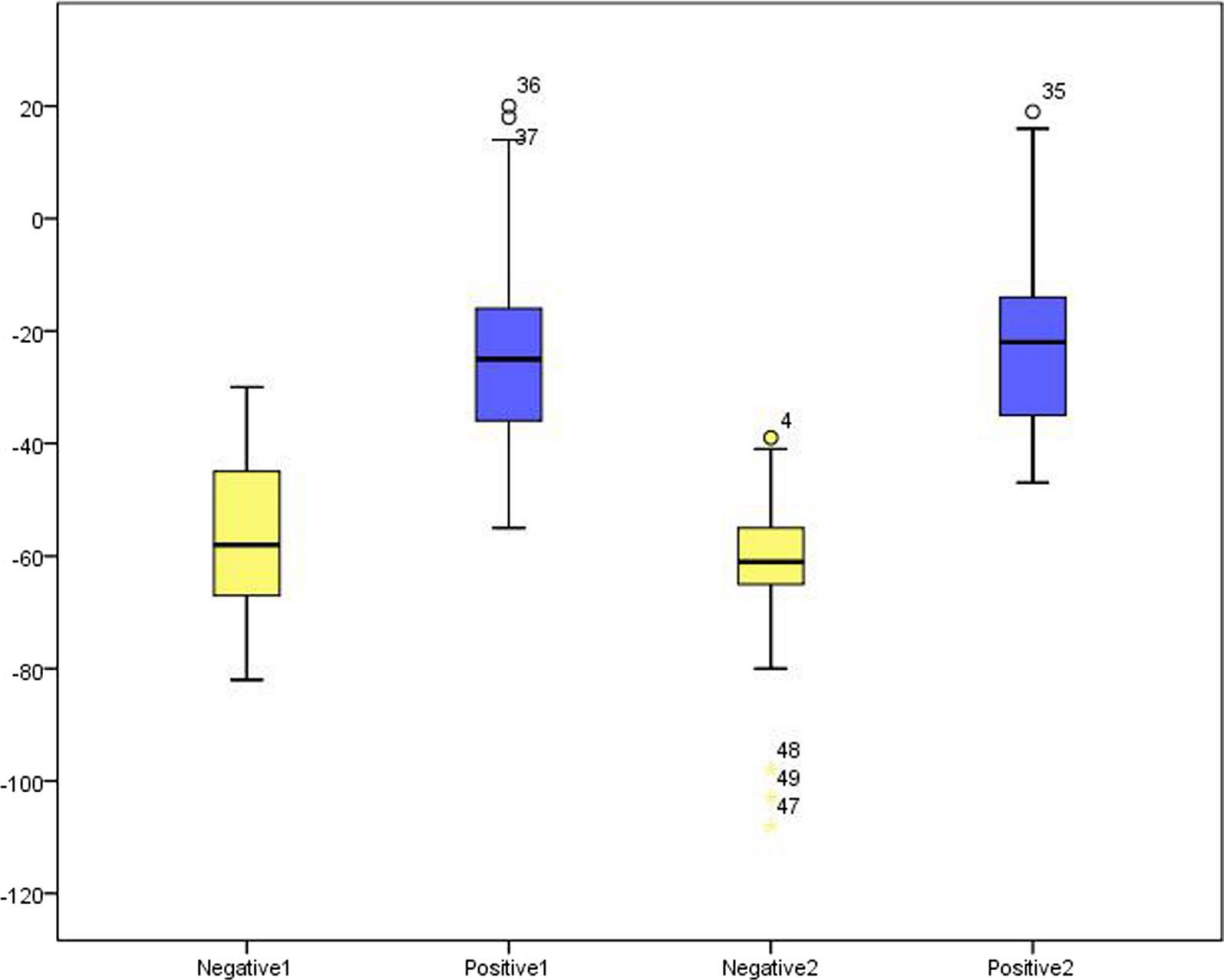
Mean CT numbers of positive and negative ELMSI, with Grade 3–4 ELMSI as a positive result and Grade 1–2 ELMSI as a negative result according to MRI; 1 and 2 in the figure represent Reader 1 and Reader 2

Table 3 shows the mean CT numbers for different grades of ELMSI on DECT VNCa images. Based on the findings of the two readers, we found that the higher the grade of ELMSI, the higher the CT numbers, and the participants with different grades of ELMSI also had significant differences in DECT VNCa images (*p* < 0.001 for both readers) (Table 3). According to the MRI results, Grade 1–2 ELMSI were defined as negative and Grade 3–4 ELMSI were defined as positive, and the mean CT numbers were measured (Table 4 and Fig. 5).

**Table 3:** CT numbers of different grades of ELMSI on VNCa images based on MRI

BMLs grading	Reader 1	Reader 2	Mean CT numbers
Grade 1	− 88 ± 9 (− 105 to − 76)	− 93 ± 9 (− 110 to − 79)	− 90 ± 7 (− 103 to − 81)
Grade 2	− 56 ± 13 (− 82 to − 30)	− 60 ± 9 (− 80 to − 39)	− 58 ± 8 (− 74 to − 42)
Grade 3	− 33 ± 9 (− 49 to − 17)	− 32 ± 9 (− 45 to − 17)	− 32 ± 7 (− 43 to − 17)
Grade 4	− 18 ± 17 (− 55 to 20)	− 14 ± 17 (− 43 to 19)	− 14 ± 17 (− 40 to 4)
*p* value	< .001	< .001	< .001

Data are expressed as mean CT numbers ± standard deviation, derived from dual-energy VNCa images

Data in parentheses are the range

*p* value was calculated with the two-sample t test. *p* < .05 indicates significant difference

**Table 4:** CT number analysis of positive and negative regions of ELMSI by MRI

Variable	Reader 1	Reader 2
Negative	− 62.1 ± 17.8	− 66.7 ± 16.9
Positive	− 24.0 ± 16.4	− 22.0 ± 17.4

Data are mean CT numbers ± standard deviation, derived from DECT VNCa images

**Fig. 6:**
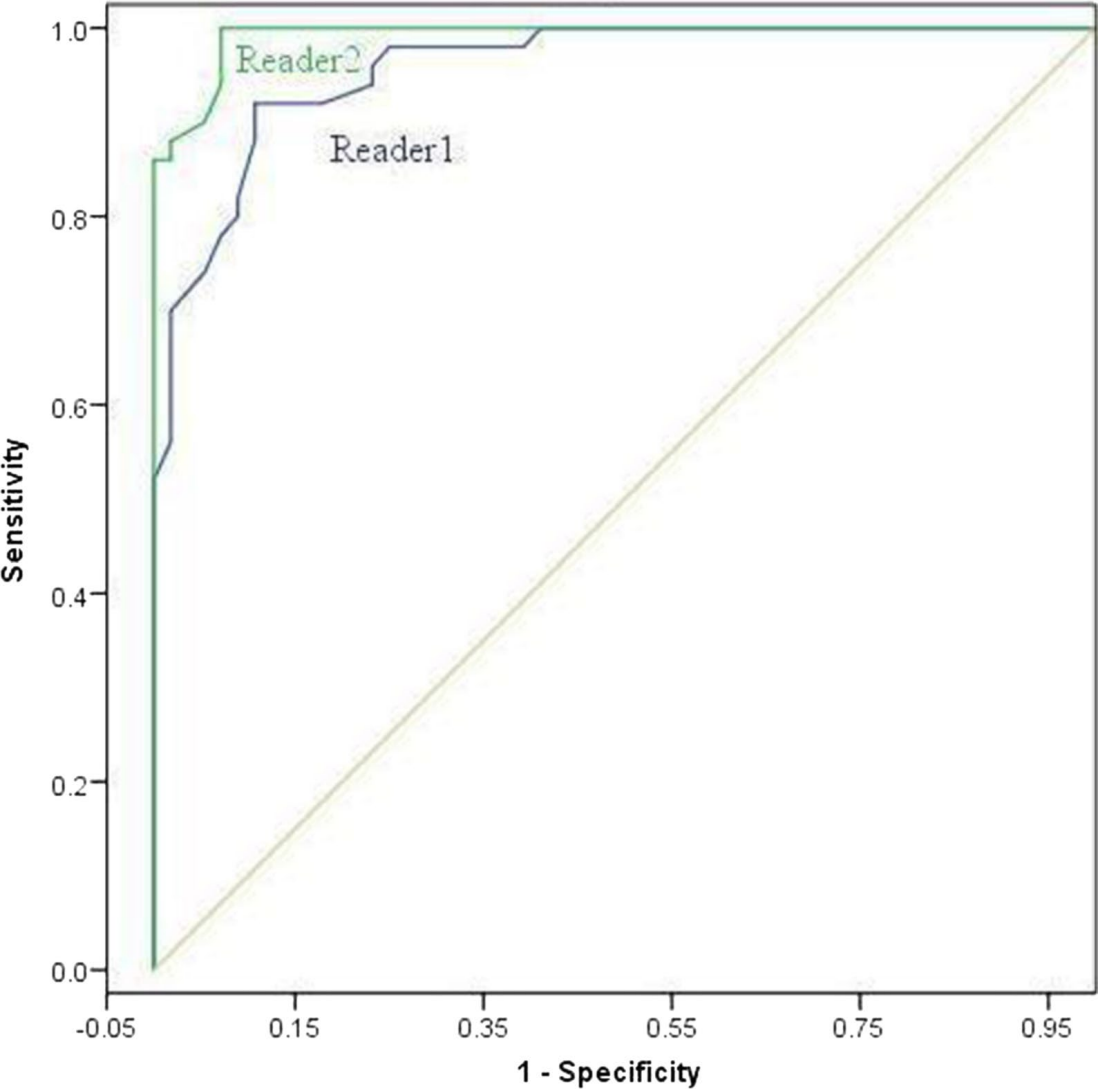
Shows ROC curves calculated from CT numbers derived from DECT VNCa images for two independent readers for the differentiation of knee joint osteoarthritis with and without ELMSI. The area under the curve was 0.961 for Reader 1 and 0.992 for Reader 2 (Blue represents Reader 1 and green represents Reader 2)

We also analyzed different grades of ELMSI on the DECT VNCa image by ROC curve analysis. The area under the curve for Readers 1 and 2 was 0.961 (95% CI 0.93, 0.99) and 0.992 (95% CI 0.98, 1.00), respectively. The results are shown in Table 5 and Fig. 6. The cut-off values for Reader 1 and Reader 2 on the ROC were -41HU and -47HU, and the sensitivity and specificity were 87.8% and 99.5% and 98.0% and 99.5%, respectively (Table 5).

**Table 5:** The diagnostic performance of VNCa images in ELMSI based on MRI

Variable	Reader 1	Reader 2
Area under the ROC curve	0.961 (0.93, 0.99)	0.992 (0.98, 1.00)
Cut-off value	− 41	− 47
Sensitivity (%)	87.8 (74.5, 94.9)	98.0 (87.8, 99.9)
Specificity (%)	99.5 (97.9, 99.9)	99.5 (97.9, 99.9)

Data in parentheses are 95% confidence intervals

## Discussion

The results of this study showed that DECT VNCa imaging has an encouragingly high agreement with MRI for the detection of ELMSI in knee joint OA. ELMSI can be qualitatively and quantitatively analyzed by ROI-based CT numbers measurements. We also found that the CT numbers of bone marrow are closely related to the grade of ELMSI. There were statistically significant differences in CT number of the bone marrow between the four grades of ELMSI.

The results of our qualitative analysis showed that VNCa images have excellent performance for the detection of ELMSI changes in OA. Stated simply, calcium is subtracted from the DECT during post-processing to obtain the VNCa images, allowing the identification of increased non-specific CT numbers. Current ELMSI research mostly involves trauma-related ELMSI, and less information exists about arthritis-related ELMSI in chronic diseases such as OA. This may be because MRI is not the preferred method for evaluating trauma-related acute pain.

We have previously reported that DECT VNCa has good diagnostic performance in the detection of carpal joint contusion and is a potential alternative scanning method for patients with contraindications to MRI [37]. Currently, most investigators use VNCa images to detect trauma-associated ELMSI, and qualitative and quantitative analysis of DECT VNCa images has had high diagnostic performance for these lesions [38–40]. Some investigators have reported good diagnostic performance of VNCa in detecting the infiltration pattern of multiple myeloma-associated ELMSI. Notably, VNCa technology has been applied for the detection of ELMSI in the sacroiliac joint and good correlation was obtained [41]. Our data show that quantitative analysis of DECT VNCa images is effective for the diagnosis of ELMSI in patients with knee OA. The difference in CT numbers between ELMSI-positive and ELMSI-negative regions is important for determining the quantitative and qualitative diagnostic results of DECT VNCa imaging [39, 41].

In our study, this significant difference enabled us to detect ELMSI on VNCa images. We measured the attenuation of bone marrow on VNCa images reconstructed from DECT and found a significantly higher attenuation in regions that were positive for ELMSI than in those that were negative for ELMSI on MRI. However, in several cases, MRI-positive ELMSI were not detected on qualitative analysis of the VNCa images. When we suspect ELMSI in DECT VNCa images, the diagnostic efficiency can be increased by measuring CT numbers [42, 43]. We also used a ROC curve cut-off value to analyze suspected ELMSI. The cut-off value greatly improved the sensitivity and specificity of ELMSI detection. The value of Rel.CM plays an important role in post-processing VNCa images and influences the qualitative and quantitative evaluation of ELMSI. We used the multi-plane reconstruction image to measure the CT numbers of the bone marrow. Besides the bone marrow, knee OA also involves ligaments, menisci, and other structures. Various DECT post-processing technologies can be used to better display ligaments, menisci, bursae, and other structures, which greatly improves its diagnostic performance for the disease [44]. Our data show that VNCa technology may be an alternative method for detecting ELMSI in patients with contraindications to MRI. DECT scanning speed is fast and related to the convenience of image post-processing. Therefore, VNCa post-processing is of great significance for our diagnosis of OA-related ELMSI.

Our study has several limitations. First, the number of participants was small; further study with a larger sample size is needed. VNCa algorithm is unable to show mild ELMSI directly adjacent to cortical bone; this is due to incomplete masking of the cortex and spatial averaging. Third, because the DECT post-processing image settings required multiple parameters and the selection of parameters greatly impacted the ELMSI image display, we used MRI as the reference standard to detect ELMSI, so small ELMSI may not be detected on VNCa images. Future studies should include a comparison of multiple selected parameters. Fourth, MRI was used as the reference standard because of the limited pathological reference standards available.

In summary, DECT VNCa images have good diagnostic performance for qualitative and quantitative analysis of knee OA-related ELMSI. DECT VNCa images may play an important role in the diagnosis of acute pain related to OA-ELMSI.

## Data Availability

Due to the nature of this research, participants of this study did not agree for their data to be shared publicly, so supporting data are not available.

## Abbreviations

BML: Bone marrow lesion
CT: Computed tomography
DECT: Dual-energy computed tomography
ELMSI: Edema like marrow signal intensity
MRI: Magnetic resonance imaging
NPV: Negative predictive value
OA: Osteoarthritis
PPV: Positive predictive value
ROC: Receiver operating characteristic
ROI: Region of interest
VNCa: Virtual non-calcium
